# Interactions at sea: on the microbiome life-cycle and biogeochemical processes

**DOI:** 10.1007/s40656-025-00687-1

**Published:** 2025-08-18

**Authors:** Tamar Schneider

**Affiliations:** The Open University of Israel, Ra’anana, Israel

**Keywords:** Phycosphere, Phytoplankton, Microbiome, Interactions, Microbial life cycle, Biogeochemical cycle

## Abstract

The marine phycosphere is a microscale mucosal region of microbiomes surrounding a phytoplankton cell. The phycosphere (analogous to the terrestrial rhizosphere) is where microbial interactions navigate the biochemistry of the sea. The study of this microsphere deals with the causal relation enigma between two spatiotemporal scales: the micro-communal interactions and the macro-level of the biogeochemical cycles (Stocker, *Science*, *338*(6107), 628-633, 2012); Segev et al., *eLife*, 5, e17473, 2016; Seymour et al., *Nature Microbiology* 2, Article 17065, 2017). This study of communities and ecosystems looks at metabolic interactions and interdependence relations, not focusing on biodiversity as the object of study. Following marine microbial ecology, an epistemic view of interactions and inter-communal relations seems to take the bulk of consideration. In this paper, I ask what it is about the sea that promotes an interactionist epistemic framework that is different than other fields in microbial ecology. Using Helen Longino’s interactionist ontology (2020, 2021), I ask whether the sea presents a unique epistemic framework focusing on understanding interactions and interdependence. I look into the insights marine environmental studies may provide to the methodological and conceptual challenges in understanding microbial ecological stability and life cycles. By paralleling marine and soil microbial ecology, I highlight the distinct features of the water column that offer a unique epistemic and methodological framework focused on interactions and interdependence. Exploring microbial ecology at sea, I detail its epistemic advantages in shaping an interactionist theoretical and conceptual framework.

**Supplementary Information:**

The online version contains supplementary material available at 10.1007/s40656-025-00687-1.

## Introduction

The study of microbial oceanography integrates microbiology, ecology, and ocean studies. As Stephan Helmreich points out, the term “suggests that microbes are not just in the sea but, in an important sense, are the sea” (Helmreich, 2023, p. 2). The marine phycosphere is a microscopic mucosal region of microbiomes (i.e., microbial communities) surrounding a phytoplankton cell. The phycosphere (similar to the terrestrial rhizosphere) is where microbial interactions navigate the biochemistry of the sea. Phytoplankton is microalgae that, like plants, obtain their energy through photosynthesis, producing oxygen and absorbing CO2. Also known as “drifters of the sea,” they exist in large populations in the euphotic zone, the upper layer of the water column also called the epipelagic zone, where sunlight penetrates and photosynthesis can occur. These organisms represent a diverse mix of eukaryotic species, such as plant-like algae and calcium-shell algae known as coccolithophores, as well as prokaryotic cyanobacteria referred to as blue-green algae. These eukaryotic and prokaryotic autotrophs disperse in the water and share their close and intimate environment with other heterotrophic organisms, such as bacteria and archaea, forming ecological communities that drive oceanic and Earth’s biogeochemical cycles.

Phytoplankton and their associated microbial communities are organized in patches across vast areas of the ocean. These patches mutually influence each other’s molecular environments and affect patterns of microbial dispersal and succession (Stocker, 2012; Datta et al., 2016; Baquero 2021; Ebrahimi et al., 2022; Muratore et al., 2022; Schwartzman et al., 2022; Daniels et al., 2023; Lipsman et al., 2024). The metabolic interactions within and between these microscopic communities drive large-scale biochemical processes, which are significantly impacted by global ecological conditions (Behrenfeld et al., 2006; Karl, 2007; Lehahn et al., 2014; Widder et al., 2016). For example, algal blooms, which can result from elevated temperatures, act as mass producers while simultaneously increasing metabolic interactions that may harm surrounding organisms (Karl, 2007; Lehahn et al., 2014; Segev et al., 2016; Lipsman et al. preprint).

Given these connections at both micro and macro levels, microbial oceanography emerges as an interdisciplinary field. It encompasses the study of global environmental conditions in water and their influence on the stability of microbial communities, as well as examining complex mechanisms of microbial metabolic interactions and their effects on global biogeochemical cycles. Consequently, one primary challenge in marine microbiology is to develop a theory that explains the bi-directionality of causal relationships between these two scales. Therefore, studying the microscale also involves exploring biogeochemical cycles and addressing the tension in spatiotemporal scales of i-directional causality between microbial interactions at the individual or micro-communal level and the macro-level of global environmental dynamics (Stocker 2012; Segev et al., 2016; Seymour et al., 2017).

In this paper, I explore the unique features of the sea that attract marine microbiologists to study microbial interactions, in addition to the technological development of metagenomics, which addresses observational and classification challenges in microbiology. Examining interactions as both ontological and epistemic subjects, I draw on Helen Longino’s ontology of interactions, conceptualizing them as a mutual exchange between individuals (Longino, 2020, 2021). Longino’s interactionist perspective examines human behavioral studies, offering an onto-epistemic viewpoint where interactions are the central focus—not the individuals themselves, but the mutual exchanges between them and their environmental and social contexts. Building on this Longinian framework, I argue for its fruitfulness in studying marine microbiology, which emphasizes microbial interactions as the primary focus. By paralleling marine and soil microbial ecology, I highlight the distinct features of the water column that offer a unique epistemic and methodological framework focused on interactions and interdependence. Exploring microbial ecology at sea, I detail its epistemic advantages in shaping the interactionist theoretical and conceptual framework.

Starting with a discussion on phytoplankton and bacterial interactions through Helen Longino’s interactionist ontology, I explain why this perspective is more appealing to ecological and marine microbiologists than an individualist perspective. Then, I elaborate on the unique marine features compared to those in the soil that require an interaction-centered investigation. Here, I demonstrate how this interaction-centered study further underscores the philosophical challenges involved in studying microbial interactions, such as the distinctive characteristics of unicellular organisms within multicellular structures that connect micro- and macro-spatial-temporal levels. Lastly, I analyze methodological perspectives, including synthetic communities, elective cultures, and the direct method, addressing the challenge of developing models that accurately capture microbial interactions and their effects on both spatial and temporal aspects of the phycosphere (i.e., cycles of growth and decay). I conclude by discussing the spatial and temporal perspective of marine microbiology as grounded in an interactionist framework.

## The phycosphere and the study of interactions

It is clear from the literature that microbial interactions are the central focus of marine microbial ecology studies. Furthermore, to understand the microorganisms’ role in the biogeochemical cycles, marine microecology studies the metabolic interactions and interdependence, independent of microbial biodiversity. Such ecological perspective is unique to microbial ecology in general due to the challenges of classification and better access to metabolic interactions using molecular biology (Prosser et al., 2007; Hugenholtz et al., 2016; Mony et al., 2020; Rainey et al. 2020; Schneider, 2022, 2023). Because of classification challenges on the one hand and advanced molecular biology practices on the other hand, the complex ecological dynamic within the microbial community becomes the object (or subject) of study. Microbiologists have better access to metabolic processes, protein changes, and non-specific genetic sequences than species classification. Therefore, the study centers on the microbial interactions within and between communities and their mutual effect on the environment (Doolittle & Inkpen, 2018; Rainey et al. 2020; Schneider, 2023).

What does it mean to discover patterns and explanations for microbial activity and life cycle at the community level? My question is both epistemic (or rather onto-epistemic) and methodological, examining how microbiologists frame their hypotheses and causal explanations using interaction ontology. Helen Longino’s ontological perspective of interactions discusses the importance of conceptualizing the object of study as part of operationalizing the phenomenon we wish to examine. The first step in establishing a certain methodology is to communicate the ontology of the object of study. This communication is in the form of asking questions: what is the thing being investigated as the assumed causal actor within the system? How to describe it? What would be the best way to study this thing? In isolation or within a system? How to better understand the elements within a system? These questions concern the object (or subject) of the study and guide the processes of inquiry from hypothesis to experimental design and the interpretation of results (Longino, 1979, 2020, 2021). Microbiology deals with two central questions that are reiterated: who are these tiny creatures? And what do they do?

Longino’s analysis shows how the background beliefs about the object of study vary between what Longino terms “individualist” and “interactionist” ontology (Longino, 1979, 2002, 2020). This distinction helps imagine different paths of scientific investigation, one that wishes to research individuals and the other examines their interactions. Thus, the background beliefs regarding the object of study play a role in framing causal explanations and hypotheses. For example, causal explanations in certain specific cases in microbial ecology can rely on an individualist conceptualization, such as the assumption that microbial activity is the result of the microbial possession of certain traits acquired in the processes of evolution by natural selection. Thus, investigating microbial species, genealogy, and evolutionary patterns can explain their behavior, dispersal, and abundance in certain places. The object of study in these practices is the individual cell/group/strain/species, and the traits belonging to this individual simplify the methodology of understanding the individual’s behavior and activity.

However, in the majority of cases in microbial ecology, this conceptualization is dubious at most or entirely unavailable for three main reasons: first, the inaccessible practice and concepts of classification at the microscale level (Prosser et al., 2007; Hugenholtz, 2016; Schneider, 2022). Second, the microbial horizontal gene transfers and mobile genetic elements challenge the genealogical tree, making it look more like a forest or entangled bush (Doolittle, 2013). Lastly, adding to the classification and ordering of microbial genealogy challenges is the unique microbial communal ability to modify their microscale environment and their properties (whether by gene activation switch or genetic modification) accordingly also depending on, and interacting with, their environmental domain (Hemmerlie et al., 2022; Daniels et al., 2023). Therefore, in many studies of microbial ecology, the aim is to research and understand microbial molecular and metabolic activities (e.g., molecular signaling, LGT, motility, chemotaxis, and virulence) in terms of productivity (biogeochemical processes) independent of their taxa and classification.

In traditional evolutionary thinking, interactions are dictated by the individuals with their traits shaped by adaptation and natural selection. However, because of microbial classification challenges, on the one hand, and accessibility to their metabolic interactions on the other hand, there is a considerable and growing literature discussing the epistemic framework of processes and interactions in evolution (Dupré & O’Malley, 2009; Doolittle & Booth, 2017; Lloyd & Wade, 2019; Suárez & Triviño, 2020). John Dupré and Maureen O’Malley (2009) challenged the individualist view in evolution, arguing for the importance of inter-organismic collaboration in defining living entities. This view considers the ecological metabolic interactions among lineage-forming microbes (e.g. viruses or MGE) as significant in evolutionary processes (Dupré & O’Malley, 2009; O’Malley, 2016). Another example of thinking about individuals and interactions in evolution is the model of “It’s The Song Not The Singer (ITSNTS)” suggested by Ford Doolittle and colleagues, arguing that selection works on the metabolic pathways, not on individual microbial traits (Doolittle & Booth, 2018; Doolittle & Inkpen, 2018). This perspective points to patterns of interactions within the microbial community as the system of interactions.

Adding an onto-epistemic view to this body of work, I discuss Longino’s ontology of interaction and the differences in background beliefs about the object of study between individualist and interactionist ontology (Longino, 2020). Then, I elaborate on these two ontologies, showing why the interactionist framework is epistemically more attractive to microbiologists exploring the oceanic phycosphere.

### Longino’s background beliefs and ontology of interactions

Longino’s analysis explores two foundational perspectives on studying complex phenomena: the individualist and interactionist viewpoints (Longino, 2020). The individualist perspective regards the individual as the primary object of study, seeing interactions as outcomes of their internal traits. In contrast, the interactionist perspective emphasizes interactions themselves, assessing individuals through their actions and context (Longino, 2002, 2021).^1^ This view acknowledges that an individual’s behavior and abilities adapt within their interactions, highlighting their phenotypic plasticity. In essence, while individualist ontology examines causal relationships from individual traits to interactions, interactionist ontology focuses on the reciprocal relationships involving individuals and their social contexts (Longino, 2020).

The meaning of interactions in Longino’s account is taken from a definition of social interactions with modification to non-human entities as simply the agents’ mutual acting of exchange (De Jaegher et al., 2010; Longino, 2021). Interactions as the subject of study involve a distinction between individuals, their social and environmental context (relationships), their positioning within this context (relations), and their activities (interactions). For instance, a relationship could be characterized by conflict, exemplified by interactions involving the exchange of force, or by friendship, illustrated through interactions involving gift exchanges. Additionally, relations can exist without interactions, as seen when two people sit at a table engrossed in their phones. The idea of relations pertains to their positions, the concept of relationships pertains to the context of their interactions—such as friendship, romantic connections, or co-worker relationships—while the notion of interactions refers to the mutual exchanges between agents (De Jaegher et al., 2010).

Longino discusses aspects of reality in the study of human behavior examining three levels or scales. The community level of scaling up focuses on groups, while the individual level of scaling down emphasizes individuals. She argues that interaction is the missing element in this framework. Without exploring interactions, causal explanations often become reductionist, placing the individual as the primary causal point while viewing groups or communities as mere aggregates. Alternatively, they can adopt a form of holism that conceptualizes communities as individual entities. Therefore, studying interactions and their mutual effects—between the individual scaling down and the community scaling up—provides a more comprehensive causal explanation, helping to avoid both ends of this dilemma (Longino, 2021).

The study of the interaction as a middle-scale between bottom-up and top-down mirrors the micro- and macro-scale tension in the sea between the microscale individual interactions and the macro-scale ecological processes. This also addresses the tension between holism in ecology, where ecological communities or ecosystems are viewed as causal entities, and reductionism in evolution, which seeks fundamental units such as cells, genes, organisms, or species. Here, the concepts of ecological collaboration and viruses as ‘distributed individuals’ (O’Malley, 2016) or Doolittle’s selection of processes (ITSNTS) can benefit from an ontological view on interactions that conceptualizes them as the main focus of investigation, without making any metaphysical commitments to natural kinds.

Examining interactions within relational domains allows for a nuanced understanding that connects individuals and their contexts without being reductionist or holist (Longino, 2021). The social or environmental domain encompasses relationships that shape interactions, such as co-workers in a workplace or a married couple within the marital institution. These relationships influence how individuals interact and the cohesion they experience, impacting personal autonomy. Domains like marriage, family, and workplace establish social and economic norms that dictate the nature of relationships—ranging from care, collegial, rivalry, or alienation (Longino, 2021). While the domain defines relationship types, the nature of interactions within can vary widely, leading to either personal growth or oppressive dynamics. Interactions in this regard connect the micro-scale of individuals with the macro-scale of their social and environmental domain.

### The ontology of interactions in microbial ecology

Taking this ontological view to study microbial interactions, the domain is the social and environmental context such as microbial communities and their environmental niche. Here, two essential components need to be taken into consideration: one is the environmental and social context, i.e., the domain in which the interactions take place, such as the social domain of a group, community, and meta-community, and the environmental domain such as microbial patch, niche or the larger domain such as the geographic area of the phycosphere. The other is the examination of the individuals’ activities and their changing levels of autonomy/interdependence. Thus, when the examination centers on the phenomenon of interactions, the research should consider the activity or processes of mutual exchange, the type of relationships (competition, collaboration, or coordination), and the social and environmental domain (i.e., various bimolecular techniques to follow taxonomic and metabolites composition as well as micro-macro scale of physical and geographical conditions).

The causal relations between the individuals’ behavior, their interactions, and their social-environmental domain are reciprocal (or better thought of as mutual or entangled). The domains define the conditions and types of relationships, but also the interactions can change the conditions and definitions of the domain (i.e., marriage and divorce, working conditions and alike in the social example, spatial, chemical, geographical, and nutritional conditions in the case of microbial ecology). The microbial domain in the phycosphere establishes their various relationships and division of labor within microbiomes, among them, and with their algal neighbors. These domains consist of complex interactions that are interdependent and go through changes during the life cycle of the phytoplankton from early development to the picks of its bloom and then down the path of decay. The study of causal connections between the community scale and the individual scale in these bloom and bust cycles is a good example of interactions’ explanatory role in connecting these scales.

One of the lead directions of investigation conceptualizes the microbiome in the phycosphere as a bio-molecular multicellular structure similar to a biofilm (but not identical). This marine microscopic multicellular structure is not conceptualized as an individual, it is transient and composed of microbial interactions that go through changes as they change their close and immediate environment. For example, as a result of the microbial productivity of minerals, they create a rich environment for the phytoplankton to grow and prosper. Continuing this growth, the ratio between young, aging, and dead phytoplankton cells changes. Young and aging cells release different types of molecules. With the growth of the population, the molecular environment changes from lightweight molecules released by young cells to heavy and less diffusible molecules released by old and lysed cells (Seymour et al., 2017; Schwartzman et al., 2022). 2 The nutrient change leads to changes in the microbial behavior and metabolic pathways, changing their interactions. Microbiologists refer to this microbial ability to change metabolic pathways as phenotypic heterogeneity, viewed as an ecological adaptation mechanism that allows microbes to change their activity in coordination with their degrading sources of nutrients (Schwartzman et al., 2022; Hemmerle et al., 2022).

Furthermore, as these microscopic processes are on a spatial and temporal continuum, it seems that the microbial life cycles parallel a microbiome-reoccurring cycle responsible for the bloom and decaying cycles in the phycosphere (Karl, 2007; Datta et al., 2016; Ebrahimi et al., 2022; Muratore et al., 2022; Schwartzman et al., 2022). The entangled life ontology of microbes and microbiomes creates some form of multicellular “life cycle”. In these relationships, microbial phenotypic heterogeneity is an eco-evolutionary mechanism enabling the succession and reproducibility of the communal structure as part of the biogeochemical cycle. Thus, a conceptualization of the microbiome as a reoccurring multicellular structure with a cycle parallel to the biogeochemical cycle is based on the understanding of microbial ability to coordinate gene activation and transmission. 3 This ontology of causal relations follows the interactions and interdependencies between the individual microbes, their interactions, and the environment.

As the individual microbe is the leading actor in this network, it becomes clear that the interactions involving these microbes are key to uncovering the mechanisms constraining and guiding their behavior into different forms of coordination, collaboration, and competition (Griesemer & Shavit, 2023). Therefore, many of the marine microbiology labs take the challenge of conceptualizing microbial interactions as the subject of study, looking for creative ways of operationalizing interactions as part of their investigation. I discuss this further in the next two sections examining the unique features of marine biology that favor the ontology of interactions. First, I examine the water column as an environmental domain with its unique characteristics compared to the soil. Then, I look at the unique methodology addressing the challenge of accurately capturing microbial interactions and their effects on the phycosphere macroscale.

## The water column and the microbial soup – the micro and macro scale tension

This section examines the unique properties of the water column that shape the study’s focus on interactions. The tension between micro and macro scales is intrinsic to the study of interactions, particularly regarding the social and environmental domains. However, in the context of the sea as the environmental domain, this tension is evident through the physical characteristics of dynamic water compared to the more stable conditions found in soil. To clarify these features, I compare the marine phycosphere with the terrestrial rhizosphere, as both share complex mutualistic relationships that can be generalized on a global scale for all plants and phytoplankton. This comparison highlights the connection between micro and macro scales, which is more evident in marine environments than in soil. It underscores the mutual influences at these scales and the significance of interdisciplinary collaboration between microbiology and earth sciences (Karl, 2007; Stocker, 2012; Behrenfeld & Boss, 2014).

The phycosphere is the area where phytoplankton cells interface with the water similar to the rhizosphere area where plants’ roots interface with the soil on land. These particular microscale areas are the domain of temporal and dynamic patches of microbial communities feeding on the carbon secreted by the roots or microalgae neighbors and releasing metabolites and nutrients for the roots or microalgae to consume. Recent findings suggest that this mutualistic relationship is more complex than cross-feeding, and there are other mutual benefits such as the supply of vitamins as well as protection from pathogens (Bakker et al., 2013; Berg et al., 2014; Segev et al., 2016; Seymour et al., 2017; Ling, 2022; Hemmerlie et al., 2022; Beiralas et al., 2023; Lipsman et al., preprint; Eliason et al., preprint).

Thus, these microscopic and dynamic relationships are a complex of mutualist, antagonist, commensal, parasitic, and competitive systems of interactions responsible for the thriving and persistence of plants on land and the phytoplankton at sea. Furthermore, these complex systems of relations are unstable and, depending on various known and unknown factors, can change from stable and balanced communities to pathological and destructive ones (Lehahn et al., 2014; Segev et al., 2016; Abada et al., 2023). Therefore, the thrive and decay of plants and phytoplankton is dependent on their microbial communities, while the stability of the ongoing microbial communities’ life cycle is reciprocally dependent on the environmental conditions and the life cycle of their eukaryotic partners.

At the very basic ecological understanding, the microscale domain of the phycosphere and rhizosphere facilitate mutualistic, co-evolutionary, and long-lasting eukaryotes-prokaryotes interdependence relations. Like niche constructing landscapers’ organisms influencing the habitat and availability of nutrients for other organisms in their environment (i.e., dams, mounds, nests), microbial niche construction (similar to that of earth warm affecting the soil humidity) changes the molecular composition of their immediate environment providing nutrition, vitamins, and protection to the plants and phytoplankton. Furthermore, there are indications that both phytoplankton and plant roots actively exudate specific nutrients to attract their favorite microbial species, thus actively shaping and gardening their microbiomes (Seymour et al., 2017; Ling et al., 2022).

In both land and water, the microbial metabolic interactions mutually affect their molecular environment, but with physical and chemical differences between the soil on land and the water at the upper surface of the sea. The microbial communities in both shapers are unstable, and the water column and the conditions on land affect particle movement and fusibility of nutrients, differently. The water movability and dynamic compared to the relative steadiness of the soil affects the availability and spread of nutrients as well as the need and utility for microbial motility (Seymour et al., 2017). For example, while microbial motility is abundant in the soil, attachment and detachment from particles seem to be better strategies for microbial foraging in a giant moving fluid (Seymour et al., 2017; Ebrahimi et al., 2022; Schwartzman et al., 2022). Therefore, marine microbiologists rarely sample non-attached microbes compared to the soil.

As the microbiome in the rhizosphere interacts with microbes from above ground and the bulk soil, the microbiome phycosphere mostly gathers around organic hotspots attached to the phytoplankton cells or floating in the upper levels of the sea. These ephemeral hotspots sometimes construct an extracellular matrix spreading on a large space or remain separated, floating or sinking to the bottom of the sea (Stocker, 2012; Seymour et al., 2017; Lipsman et al., preprint; Ebrahimi et al., 2022).

Surprisingly and counterintuitively, at the microscale level, organic matter is heterogeneous, spreading in a gradient in both the soil and the water. In the water column, contrary to the soup metaphor, organic matter at a microscopic level is not homogenously abundant, and nutrients are distributed heterogeneously (Stocker, 2012). The sea contains small organic particles spread out like snowflakes sinking to the bottom while shedding microscopic dissolved organic matter (DOM) (Stocker, 2012; Seymour et al., 2017). These nutrients on a microscale level do not spread homogeneously but rather bundle together with oxygen, creating microscopic hotspots attracting microbes forming communities that digest, decompose, and break down the organic matter into inorganic energy sources such as carbon, nitrogen, and phosphorous (i.e., a process called remineralization). Many of these hotspots are gathered by phytoplankton to attract productive and beneficial microbes (Stocker, 2012; Seymour et al., 2017). The DOM and organic hotspots are also ephemeral as they are constantly gathered and consumed (Stocker, 2012).

This gradient liquid ecological landscape is responsible for microbial activity and behavior where microbes are busy foraging for nutrients, determining their composition and interactions within their community and meta-community (Ebrahimi et al., 2022). Furthermore, as phytoplankton and microbes are in constant need to establish connections to withstand the physical conditions, they create these interconnected structures mutually influencing each other in close and intimate molecular environments (See the example in Sect. 2.2. on the influences of phytoplankton life cycle on the molecular composition in their environment). Thus, both the microbiome and the phytoplankton change the molecular composition of their immediate environment, which in turn changes the mutualistic nature of their interactions (Lipsman et al. preprint; Abada et al., 2023; Daniels et al., 2023).

The water’s physical and chemical conditions affect the organic and inorganic dynamics of diffusion and sinking rate, while the microbial interactions affected by this dynamic also change the organic and inorganic composition in the water through their metabolic interaction (Daniels et al., 2023). The availability of nutrients in the water can determine microbial activity in the phycosphere from commensalism, mutualism, and pathogenicity. On the microscopic scale, the mutual nature of microbial symbionts affects the prosperity and longevity of the phytoplankton population. For example, not all of the microbes in the phycosphere are heterotrophs that use organic matter for energy. Some are autotrophs competing with the phytoplankton over minerals in the water as well as products of the remineralization process (Karl, 2007). Also, some of the autotrophs within the microbiome can change their metabolic path to consume inorganic matter when the organic matter becomes scarce, adding to the competition with the phytoplankton and other autotrophs (Seymour et al., 2017).

Thus, the phenotypic characteristics of different groups within a microbiome can shift depending on the organic and inorganic environmental composition. This change can shift mutualist cross-feeding interactions into competitions, causing niche partitioning (Muratore et al., 2022; Daniels et al., 2023; Schwartzman et al., 2022). Also, as discussed above, these complex interactions change phases and activity between mutualism, competition, and antagonism depending on changes in their small and large environmental scale (Lehahn et al., 2014; Segev et al., 2016; Beiralas et al., 2023). Thus, not only do their interactions and metabolism influence their activity but also seasonal and circadian changes.

This macro-microscale complexity is reflected in microbial interactions as they play an important role in the dynamic and interchangeable marine environment. The microbial communal structures and their interdependence ignited a growing interest in studying microbial interactions and the understanding of communities as multicellular aggregates collaborating with some form of coordination and division of labor depending on the gradient of organic particles and ecological cycles, such as the diel and seasonal cycles (O’Malley, 2016; Schwartzman et al., 2022; Muratore et al., 2022). In the next section, I examine the methodological challenges of developing models that accurately capture microbial interactions and their spatial and temporal aspects of the phycosphere (i.e., cycles of growth and decay and the biogeochemical cycles).

## Scaling down and scaling up: on interactions and the Spatial and Temporal connections

The first step in understanding the phenomenon of microbial interactions is to find ways for the study to focus on processes and patterns of interactions not based on the assumption of innate constant individual traits (Doolittle & Booth, 2017). When the subject of study is the microbial species such as commonly thought in the study of a pathogen, the study looks at its traits, and genealogy, and collects aggregates patterns of its behavior. However, interactions are not objects or individuals, they are processes of mutual exchange that are also embedded within a context (Longino, 2020, 2021). Therefore, the study of oceanic microbiology includes microbial physiology and activity in concordance with changes in their immediate physical, chemical, and organismal environment. Thus, laboratory practices such as pure culture, co-culture, and synthetic communities are considered model systems to be joined with ecological field approaches. Multiple samplings from the sea are easily approached compared to sampling soil microbes because they already exist in a liquid. Thus, the direct method can be achieved with less tinkering with the growing medium (Helmreich, 2009, p. 183).

The general thought is that the open sea should be the laboratory setting, and microbiologists find creative ways to bring the lab biomolecular technology with them as their observation methods at the open sea (Karl, 2007). Despite the liquid medium, direct observation is still a challenge both in the lab and out in the wild, as the microscopic interactions are dynamic and sensitive to the physical, chemical, and biomolecule environmental conditions. Sampling technology, though immensely advanced, is still limited in the sense that it is missing the spatial and temporal perspective of the molecular environment in which the microbiome develops and evolves (Karl, 2007; Helmreich, 2009; Bakker et al., 2013; Quistadet al., 2020; Muratore et al., 2022). The practice of elective culture in the lab can give a specific perspective on the hypothesized developmental process but is limited in understanding the microbial interactions in nature and over time. Thus, marine laboratories around the world use creative methods to pace together molecular microscale samplings like stills pictures turned into a stop motion movie of phytoplankton and microbiome life cycles (Seymour et al., 2017; Ling et al., 2022).

To complement the laboratory and in-vivo study of phytoplankton and microbiome interactions, the study also involves interdisciplinary collaboration with marine and earth sciences. Considering the phytoplankton essential role in Earth’s biogeochemical processes this microsphere is also influenced by global climate and ecological trends. Therefore, microbial oceanography involves ecological measurements and observation on a large scale. Studying the ocean’s ecological and biochemical processes follows physical and chemical measurements such as the ocean-atmosphere interactions, current trends, and the water optical properties influencing the conditions of gas concentration and temperature, as well as the spread and diffusion of the organic gradient. This also includes the biochemical sampling of proteins and metabolites to follow the rates of biomass and productivity levels (Karl, 2007; Stocker, 2012; Lehahn et al., 2014). This information is gathered with the use of innovative equipment of direct sampling during periodic cruises to obtain continuous measurements of various atmospheric and oceanographic parameters covering both diel and seasonal cycles (Karl, 2007; Behrenfeld & Boss, 2014; Lehahn et al., 2014; Widdler et al., 2016; Hackl et al., 2023).

Time is one of the key aspects that keep coming up as a major challenge in the investigation as the phycosphere activity is constrained or manifested through multiple entangled cycles. The marine microbial communities are in constant flux of change due to the processes of living, consuming, and releasing matter and gases. It is a constant movement of change and exchange of growth and decay and succession, attachment and detachment (Datta et al., 2016; Schwartzman et al., 2022). Furthermore, the many metabolic processes such as cross-feeding and other trophic interactions create biochemical processes that are part of biogeochemical cycles. Thus, the microbial life cycles, the microbiomes’ reoccurring cycles, and the phycosphere bloom and bust cycle are all entangled with different lengths of daily, monthly, seasonal, and geological cycles (Behrenfeld & Boss, 2014; Segev et al., 2016). Therefore, just as the macro and micro spatial scales are significant in the study of interactions, time scale, in terms of micro and macro cycles, is also a methodological and conceptual challenge.

Unlike the growth of a colony or synthetic community in the lab that has a beginning and ending timeline of growth, stagnation, and decay, the intertwined community consortia of these linear short-life processes within the phycosphere are on a cyclical timeframe. Depending on the duration, when looking at growth or decay, the timeline can be linear in one direction of each microbial cell, group, and community with a beginning and end. However, when thinking ecologically, after the crush of one community comes a succession of another, and the cycle continues. While in laboratory experimentation, the study usually stops at a certain point in time with stagnation or the collapse of the community, the timeline in nature does not stop. The collapse of microbial communities changes the environment and interactions establishing a path for the next cycle. Therefore, scientists need a theoretical concept of developmental, evolutionary, and ecological timeframe to capture this liner-circular time. (e.g., a circular time frame that is composed of many entangled and different short and long linear time frames).

The conceptual challenge described by this circular and linear time frame presents a need to understand both continuity and repetition at the same time. Such a time concept is similar to descriptions of traditional ways of living that are embedded in seasonal food and shelter taken from environmental Indigenous literature. A good demonstration of this idea is the story of the First Men Nanabozho and the notion of time tolled by Robin Wall Kimmerer in her book “Braiding Sweetgrass: Indigenous Wisdom, Scientific Knowledge and the Teachings of Plants”:

For in the popular way of thinking, history draws a time “line,” as if time marched in lockstep in only one direction. Some people say that time is a river into which we can step but once, as it flows in a straight path to the sea. But Nanabozho’s people know time as a circle. Time is not a river running inexorably to the sea,>but the sea itself—its tides that appear and disappear, the fog that rises to become rain in a different river. All things that were will come again.

In the way of linear time, you might hear Nanabozho’s stories as mythic lore of history, a recounting of the long-ago part and how things come to be. But in circular time, these stories are both history and prophecy, stories for a time yet to come. If time is a turning circle, there is a place where history and prophecy converge—the footprints of First Mean lie on the path behind us and on the path ahead (Kimmerer, 2013, pp 206 − 205).

As life is linear, with a starting time at birth and ending in death, this linear life is an active part of a larger and circular timeframe. Kimmerer describes the linear and the cyclical timeframe as analogous to the river and the sea. The river represents the one directionality of time and the sea represents the spirality of time. Looking at the phycosphere, the individual microscopic cells grow in a short linear timeline, but they interact with each other and with particles in their environment on a continuum and ongoing circular time frame.

The sea of interactions is composed of tiny multicellular structures with a timeline that also interacts with other such structures in its environment participating in global biogeochemical processes (Datta et al., 2016; Ebrahimi et al., 2022). In this small to global entanglement, every individual reacting to their environment can change their behavior and metabolic activity. The reciprocity and mutuality of interactions of many linear timelines of microbes interacting in their social and environmental domains create these circular small and large nets that maintain living. In a way, such circularity and linearity can also be found in a multicellular organism’s physiology.

Studies of the global scale and the microscale both aim to predict and understand phenomena such as the ocean ecology and its biogeochemical cycling, seasonal and harmful algal bloom, ocean pollution, and other effects of climate change (Behrenfeld & Boss, 2014; Lehahn et al., 2014; Segev et al., 2016; Lipsman et al., preprint). The interdisciplinary path helps to mitigate the macro-microscale causal connections where tests and samples of variables are taken from the same site in addition to model systems of pure cultures and synthetic communities in the lab. The presupposition regarding the scales’ causal connections is that the large global ecosystems have emergent properties manifesting through the mediation of processes of transformation of matter and energy by the microscopic microbial communities and their life cycles. The microbial activity of energy and nutrient transformation is the driving engine of the biochemical processes, but these activities are mediated and facilitated by a plethora of interactions that are also interdependent sharing their environmental domain.

Such bi-directionality in terms of the ecological food web and the biogeochemical processes are circular and spiraling in concordance with seasonal and circadian cycles. This description of reality conceptualizes the spatial and temporal dynamics between individuals and collectives through mutual exchange and environmental modifications. The spatial tension between macro and microscale mutual influence and interdependence can be conceptualized within the cyclical timeframe. For example, when trying to understand and predict harmful algal bloom, the linear timeframe of starting, growing, and collapsing is composed of many different circular metabolic processes of interactions. Interactions are events susceptive to changes in their environment, such as diel and seasonal changes or changes in resources. Thus, when thinking of cyclical times of interactions, one can imagine the diel cycle, hormonal cycle, biogeochemical short and long cycle, growth and decay cycle, and inter-generational cycle. Each cycle influences the course of interactions to repeat itself with minor to large changes.

## Conclusion

In examining studies of the phycosphere, which includes a large biomass of prokaryotic, archaea, and eukaryotic microbes, I argue that microbiologists focused on interactions should adopt an interaction-based perspective in their approach and methodology. Reflecting on the water column compared to the soil—both physical and chemical—such as nutrient distribution, dissolution, and sedimentation, I demonstrated the conditions for the epistemic bidirectional link between microscale interactions and macroscale phenomena. I then, explored the temporal and spatial characteristics of marine microbial ecosystems as they arise from methodologies studying the activities of microbial communities. These activities are driven by processes such as dispersal, colonization, and foraging, which constantly exchange matter and energy flow. Finally, applying Longino’s interaction ontology to microbiology, I propose a methodological framework that studies interactions across various temporal and spatial cycles, including the microbial life cycle, microbiome cycles, physical cycles (such as diel and seasonal changes), and biogeochemical cycles.

I outline three key methodological implications for studying interactions in oceanic microbiology. First, it’s essential to focus on the interdependence of microbial cells and groups, as these interactions influence their autonomy. As microbes can exist independently or form multicellular structures, investigating the mechanisms driving this interdependence in oceanic environments is crucial. Second, we should examine the bidirectional constraints of social and environmental domains on microbial activities. Microbial metabolic activities not only alter their environment through the transformation of matter and energy but also face environmental changes that impact those activities. Understanding these mutual influences is important. Finally, it’s vital to explore the interconnected spatial and temporal cycles between interacting microbes and their environment over ecological and geological time. This involves recognizing the interconnected spatial and temporal cycles, such as microbial life cycles entangled with the cycles of microbial communities assembling and the biogeochemical cycles.

Longino’s ontology of interactions identifies three scales of reality in the study of human behavior (Longino, 2021). The downscaling of individuals, the upscaling of collective aggregates, and the intermediate scaling of interactions establish complex dynamics within communities (Longino, 2021). My discussion on the interactionist perspective in marine microbiology adds the aspect of spatial and temporal connections as cyclical continuities. This approach underscores the role of time in Longino’s view of interactions. When we examine the micro and macro scale connections through a circular timeframe that includes diel, seasonal, and annual cycles, we can perceive the linear timeline of individuals and collectives as interwoven with circular interactions in a social and historical context. This suggests that interactions can be analyzed within circular or spiral-like generational connections. The generational perspective links the past and future at the individual level, such as family and heritage, and the broader social and environmental domains, including political and geographical contexts. Consequently, the interaction perspective examines pathways that influence future conditions while being informed by the past, allowing for the possibility of navigating present circumstances in various directions.

## Electronic supplementary material

Below is the link to the electronic supplementary material.

Supplementary Material 1

## Footnotes

1 Longino presents interaction ontology as a form of ontological pluralism, arguing that interactions should be as fundamental to analysis as individuals and their properties. Thus, this view of pluralism is not driven by reductionism or unification goals (Longino, 2020). She also notes that individualist and interactionist perspectives are not mutually exclusive and center on different aspects of the states of affairs: the former focuses on individuals as the object of study, while the latter centers on the events of interactions among these individuals (Longino, 2021).

2 Young phytoplankton release soluble and unstable lightweight molecules, such as amino acids, carbohydrates, sugar alcohols, and organic acids. In contrast, long-lived populations with high cell lysis rates will release heavy molecules such as polysaccharides, proteins, nucleic acids, and lipids. These heavy molecules tend to linger and accumulate and change the microbiome’s immediate surroundings of nutrients (Seymour et al., 2017; Schwartzman et al., 2022).

3 The phrase “reoccurring multicellular structure” describes the microbiome, maintaining the entangled and interdependent aspect of microbial communities without the ontological commitment of definitions such as individuals (organism or community). Thus, centering on interactions, the “structure” is less ontologically committing than the “individual,” but nevertheless it is part of an ecological biogeochemical cycle, and, therefore, is a reoccurring structure. The term “structure” (similar to the song metaphor in the ITSNTS model) refers to the taxonomy that enables the continuation of the cycles of biochemical processes without the specific microbial taxonomic composition, which is redundant and interchangeable in these cases. I thank the anonymous reviewer for their help in this important clarification.
