# Supplementary material for: Interactions at sea: on the microbiome life-cycle and biogeochemical processes
Source: Hist Philos Life Sci. 2025 Aug 18;47(3):41. doi: 10.1007/s40656-025-00687-1 (PMC12361257; doi:10.1007/s40656-025-00687-1)
Supplement: Supplementary file 1 — Supplementary Material 1 [file 40656_2025_687_MOESM1_ESM.docx]

**Glossary:**

**Productivity and producers**: productivity in ecology refers to the transformation of energy to biomass by processes such as photosynthesis, chemosynthetics, respiration, and metabolism. Organisms divide into primary producers that use energy sources such as light or minerals as their energy source and secondary producers that use organic matter synthesized by primary producers.

- Any biomass building and growth process is done by this exchange of matter and energy between organisms and their environment.
- These exchange processes that change the immediate environment also change the large global environment by creating a chain of reactions called the biogeochemical cycle.

**Autotrophs**: also known as primary producers, are organisms that use sunlight or chemical energy to synthesize inorganic compounds into complex organic molecules such as glucose, carbohydrates, lipids, and protein.

- Depending on the energy sources, primary producers perform photosynthesis using sunlight or chemosynthesis using inorganic molecules.
- Primary producers use energy to consume minerals and release gasses and organic compounds as waste, also called metabolites.

**Heterotrophs**: also known as secondary producers, are organisms that use organic carbon synthesized by primary producers as their energy sources.

- Primary producers consume organic matter and release gasses, organic and inorganic compounds as waste, also called metabolites.

**Lateral Gene Transfer (LGT)**: also known as horizontal gene transfer, is the transmission of genetic material between organisms, not in the familiar form, from parents to offspring through reproduction.

**Mobile Genetic Elements (MGE)**: are genetic material that can move around within or between cells, species, and kingdoms. Different types of such elements vary from transposons, plasmids, and integrons.

**Cross-feeding interactions**: the exchange of metabolites between microbial groups and microbiomes. This exchange is when microbes consume secreted metabolites from neighboring microbes. The microbiome appears to exhibit a complex network of such exchange of metabolites among microbial groups occupying the same niche.

**Interactions** refer to a process or event of mutual exchange taken from a definition of social interactions with modification to non-human entities as simply the agents’ mutual acting of exchange.

**Relationship** refers to the characterization of the social context where interactions take place. For example, in an intimate relationship, the interactions can be of different types of mutual exchange. In parasitic relationships, the interactions can be of different types of mutual exchange.

**Relations** refer to the different positions of the agents to each other (such as spatial or temporal relations). Relations can also characterize the nature of interactions such as relations of competition or collaborations. In this sense, we can think about interactions as a mutual exchange between individuals that are in some form of relations. Or describe spatial and temporal relations between individuals that are not interacting.
